# PCR and ELISA *vis-à-vis* Microscopy for Detection of Bovine Anaplasmosis: A Study on Associated Risk of an Upcoming Problem in North India

**DOI:** 10.1155/2015/352519

**Published:** 2015-02-25

**Authors:** Amrita Sharma, L. D. Singla, Paramjit Kaur, M. S. Bal

**Affiliations:** Department of Veterinary Parasitology, College of Veterinary Sciences, Guru Angad Dev Veterinary and Animal Sciences University, Ludhiana, Punjab 141004, India

## Abstract

This investigation demonstrates the status of bovine anaplasmosis caused by *A. marginale* in bovines from Submountain and Undulating Zone of Punjab. Out of 184 suspected animals, 25 (19.51%), 47 (31.71%), and 78 (68.75%) were positive by microscopy, indirect ELISA, and PCR assay, respectively. The microscopy showed 29% sensitivity and 99% specificity, while ELISA showed 32% sensitivity and 79% specificity in concordance with PCR assay. Five false negative samples by msp1*β* PCR were reconfirmed for *Anaplasma* spp. targeting 16S rRNA gene. The sequence analysis showed the presence for *A. marginale* specific restriction site, indicating variation in the local strains of the organism resulting in no amplification with msp1*β* gene primers. Of 82 samples positive by PCR, 57 were negative by ELISA indicating lower efficacy of ELISA to detect early anaplasmosis. The assessment of risk factor with results of PCR technique indicated that cattle (Odds ratio = 2.884), particularly those of age > 1 years (Odds ratio = 2.204) of district Pathankot (Odds ratio = 3.182) of Submountain Zone (Odds ratio = 2.086), were at high risk of anaplasmosis. All three districts of Submountain Zone are at higher risk indicating the impact of biotic and abiotic factors on the incidence of disease.

## 1. Introduction

*Anaplasma marginale *(Order Rickettsiales, Family Anaplasmataceae) causes pathogenic bovine anaplasmosis [1]. The organism multiplies within the erythrocytes of the host, resulting in extravascular haemolysis and acute anaemia, morbidity, and mortality in some cases [2]. Bovine anaplasmosis occurs in tropical and subtropical areas throughout the world and the disease is a major constraint to cattle production in many countries. Punjab state located in latitude 29′′30′N–32′′32′N and longitude 73′′55′E–76′′50′E provides favourable conditions for flare-up of the disease. In North India, 20 outbreaks of bovine anaplasmosis occurred during the period of January to June 2013, out of which 5 were reported from Jammu and Kashmir, 6 from West Bengal, and 9 (45%) from Punjab, indicating the threats posed on livestock by the disease (http://www.oie.int/wahis_2/public/wahid.php/Diseaseinformation/statusdetail). Thus an estimated more than 300 million of bovine population is at risk in Punjab (Livestock Census 2007). A wide variety of biological and mechanical agents are responsible for the transmission of this infection, amongst which* Boophilus microplus* is the most important vector in Punjab. After an acute phase of infection, animals may remain chronically infected carriers for years [3]. The level of parasitemia in carriers is below the threshold of detection by microscopy which has the detection limit of about 0.03 percent. The overall sensitivity of this method is 10^6^ infected erythrocytes per mL of blood. Moreover, it is time consuming and there is a need of an experienced eye to differentiate the pathogen from the related organisms including artefacts. Thus this this method is not recommended for the characterization of persistently infected cattle. Subinoculation of* A. marginale* infected erythrocytes into susceptible, splenectomized calves has been considered as the “gold standard” for detection of latent infection in cattle, but it is not practical for routine testing. Serological tests, even though developed, lack the required specificity and sensitivity for a reliable diagnosis. However, these tests for antibody detection use crude antigens obtained from partially purified* A. marginale* and thus lack the required sensitivity or specificity for a reliable diagnosis. Specific and sensitive polymerase chain reaction was developed to detect* A. marginale* DNA from animal blood and ticks which is thought to be more practical technique for diagnosis of the disease in domestic animals [4]. There were only a few previous reports on the prevalence of bovine anaplasmosis in Punjab [4] and as the propensity of tick population is higher in hilly and undulating areas, the present study targeted those areas of Punjab in particular. To the best of our knowledge, there is no previous report on the seroprevalence of* A. marginale* from Punjab. Hence, in the present investigation, bovine anaplasmosis due to* A. marginale* was comparatively evaluated by microscopy, PCR, and indirect ELISA in Submountain and Undulating Zone of Punjab to assess the level of exposure of animals in these two highly conducive zones of Punjab in relation to the risk factors associated with disease occurrence.

## 2. Materials and Methods

### 2.1. Study Area and Sampling

Punjab state is divided into five major agroclimatic zones according to their soil type, agricultural development, and precipitation and temperature indices. A representative bovine samples collection was done from March 2011 to September 2013 from the major agroclimatic zones of Punjab. Samples from hilly and undulating regions of Punjab, namely, Submountain and Undulating Zone, were selected for the study to screen the bovines with tick infestation, fever, jaundice, or anaemia for anaplasmosis. Blood (~3 mL) was drawn into anticoagulant-coated and anticoagulant-free vacutainers. Samples were processed for thin smears, nucleic acid, and sera. Data on the characteristic of sampled animals (species, age, and health status) and farms (management and location) was obtained on predesigned questionnaire during sampling.

### 2.2. Sampling Frame

To study the status of molecular and serological prevalence of the disease, the expected prevalence to be 50% with confidence limits of 95% and a desired absolute precision of 5% to collect maximum number of samples was considered. The number of samples thus calculated was adjusted for finite population and correlated with 184 samples (74 cattle and 21 buffalo; 55 cattle calves and 34 buffalo calves) collected.

### 2.3. Microscopy

From the blood samples of all the selected animals, thin blood smears were made, air dried, fixed in methyl alcohol for 2 min, and stained with working dilution of 10% Giemsa stain for 30 min. The smears were then washed with tap water to remove extra stain, air dried, and examined under oil immersion for demonstration of* A. marginale*. The blood samples were further stored at −20°C for DNA extraction.

### 2.4. Serological Study (iELISA)

Indirect ELISA (targeting gene encoding 19 kD protein) was carried out using SVANOVIR* A. marginale*-Antibody test kit. In brief, field sample, positive and negative control sera were prediluted in 1 : 100 in 1x Phosphate Buffer Saline Tween (PBST). 100 *μ*L of prediluted tested plasma was added to the selected wells and the plate was sealed and incubated at 37°C for 30 minute. After 30 min, the plate was washed four times with 1x PBST buffer. 100 *μ*L of horse reddish peroxidase conjugated with anti-bovine IgG monoclonal antibody diluted conjugate was added to all the wells. Plate was sealed and incubated at 37°C for 30 min. Again the plate was washed four times with PBST buffer. 100 *μ*L of reconstituted ABTS substrate solution was added to all the wells. The sealed plate was incubated at 25°C for 30 min at room temperature. The reaction was concluded with stop solution containing 1% SDS. The results were read in a spectrophotometer (Tecan Nano Quant Infinite M200) at 405 nm filter within 15 min of stopping the reaction. A percent positivity (PP) of negative control and samples was calculated as follows:

(1) $$\begin{array}{c} \text{PP}=\frac{\text{Mean}\;\text{OD}\;\text{value}\;\text{negative}\;\text{control}}{\text{Mean}\;\text{OD}\;\text{value}\;\text{positive}\;\text{control}}\times 100. \end{array}$$

For the interpretation of results, OD of 1.0–2.3 for positive control and PP < 20 for negative control were the criteria for test validity. Results of test samples were considered positive for PP ≥ 40 positive.

### 2.5. Polymerase Chain Reaction

For conducting the PCR assay, whole-genomic DNA was isolated from blood sample using HiPura Blood Genomic DNA Miniprep Purification Kit following the manufacturer's recommendations. Genomic DNA of* A. marginale* isolated from infected blood showing high parasitaemia was utilized as positive control. Genomic DNA was also isolated from the whole blood of infection-free day-old bovine calf and used as a negative control.

#### 2.5.1. msp1*β*PCR

DNA was extracted using HiPura Blood Genomic DNA Miniprep Purification Kit as per the protocol of the manufacturer. Amount of extracted DNA and its purity was measured at OD_260_ and ratio of OD_260_ to OD_280_, respectively. The BAP-2 and AL34S set of oligonucleotide primer was used to amplify msp1*β* gene of* A. marginale*. The nucleotide sequence of the primer [5] is as follows:

 BAP-2: 5′ GTA TGG CAC GTA GTC TTG GGA TCA 3′,
 AL34S: 5′ CAG CAG CAG CAA GAC CTT CA 3′.

The 25 *μ*L PCR reaction mixture constituted 12.5 *μ*L of KAPA2G Fast Hot Start Ready Mix (2x containing KAPA2G Fast Hot Start DNA polymerase, KAPA2G Fast Hot Start PCR buffer, 0.2 mM dNTP each, 1.5 mM MgCl_2_) with 1.5 *μ*L/0.6 *μ*M of BAP-2/AL34S primers (10 pmol) suspended in nuclease-free water with 5 *μ*L DNA template in automated thermocycler (Eppendorf, mastercycler personal) with the following programme: initial denaturation at 95°C (5 min), 30 cycles of denaturation at 95°C (1 min), annealing at 60°C (1 min), and extension at 72°C (1.5 min) with final extension at 72°C for 5 min. The amplified PCR products were separated by electrophoresis on 1% agarose gel and visualized under a UV Transilluminator for detection of 407 bp amplified product.

#### 2.5.2. Small Subunit (16S) rRNA nPCR

The conflicting samples positive by microscopy (being gold standard) and negative by msp1*β* PCR were reconfirmed by 16S rRNA nPCR. The P1 and P2 set of oligonucleotide primer was used in primary PCR cycle and P1 and P3 were used in secondary PCR cycle targeting 16S rRNA gene of* A. marginale*. The nucleotide sequence of the primer [6] is as follows:

 P1: 5′-AGAGTTTGATCCTGGCTCAG-3′,
 P2: 5′-AGCACTCATCGTTTACAGCG-3′,
 P3: 5′-GTTAAGCCCTGGTATTTCAC-3′.

The 25 *μ*L PCR reaction mixture constituted 12.5 *μ*L of KAPA2G Fast Hot Start Ready Mix (2x containing KAPA2G Fast Hot Start DNA polymerase, KAPA 2G Fast Hot Start PCR buffer, 0.2 mM dNTP each, 1.5 mM MgCl_2_) with 1.5 *μ*L/0.6 *μ*M of P1-P2 (or P1–P3 for nested) primers (10 pmol) suspended in nuclease-free water with 5 *μ*L of DNA template (or 2 *μ*L of Primary PCR product) in automated thermocycler (Eppendorf, mastercycler personal) with the following programme: initial denaturation at 95°C (5 min), 30 cycles of denaturation at 95°C (1 min), annealing at 57°C (1 min), and extension at 72°C (1.5 min) with final extension at 72°C (5 min). The amplified PCR products were separated by electrophoresis on 1% agarose gel and visualized under a UV Transilluminator for detection of 564 bp amplified product.

### 2.6. Analysis of Nucleotide Sequence

The amplicons were custom sequenced from Xcelris Genomics, Ahmedabad, India. The nucleotide sequences were subjected to BLASTn analysis [7] for determining the similarity with the sequences present in the nucleotide database. The nucleotide sequence alignment was studies by ClustalW software.

### 2.7. Statistical Analysis

Chi-square test was employed to relate the association of prevalence of infection with the different low lying areas and different groups of dairy animals under study on the basis of species and age. Win Episcope 2.0 software was applied to evaluate the agreement between the results revealed by different diagnostic tests used to detect* A. marginale* in this study. The risk factors were evaluated by calculation of Odd's ratio for all possible two way interactions. To identify goodness of fit, the observed versus predicted values (residual statistics) were derived by Win Episcope 2.0 software.

## 3. Result and Discussion

**Figure 1:**
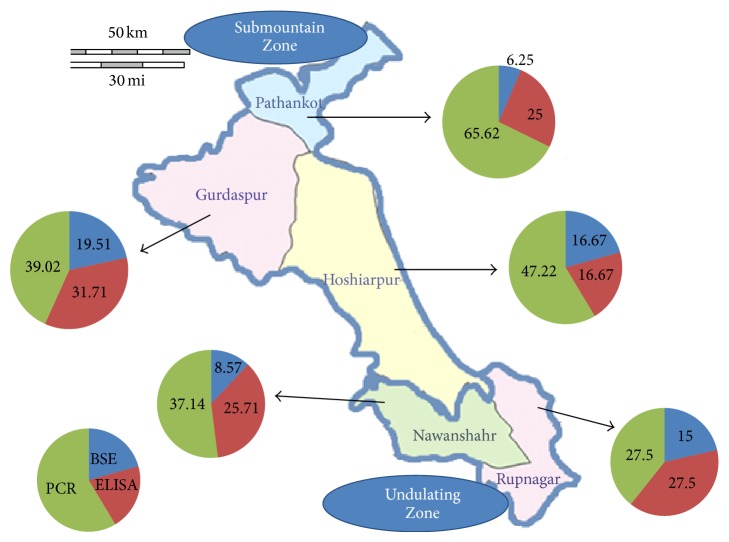
Map of Submountain and Undulatng Zone of Punjab indicating the incidence of* A*.* marginale* among different districts under study as diagnosed by Blood Smear Examination (BSE), Enzyme Linked Immunosorbent Assay (ELISA), and Polymerase Chain Reaction (PCR).

In the current study, the occurrence of* A. marginale* organisms, anti-*A. marginale* IgG antibodies, and* A. marginale* DNA in the blood of bovines from Submountain Zone and Undulating Zone of Punjab was investigated by microscopy, indirect ELISA, and msp1*β* PCR, respectively. The study results depicted high frequency of this rickettsial organism among the ruminants in the region studied. PCR revealed incidence of* A. marginale* significantly highest in District Pathankot (65.62%) (95% CI = 51.43–79.81%) of Submountain Zone and lowest in Nawanshahr (27.50%) (95% CI = 15.56–39.43%) of Undulating Zone (Table 1). The overall prevalence of* A. marginale* by msp1*β* PCR was 42.39% (95% CI = 36.23–48.54%). The difference in the prevalence did not vary significantly among various districts under study as diagnosed by microscopy and ELISA. However, both tests showed highest incidence of anaplasmosis in district Gurdaspur of Submountain Zone (19.51%, 31.71%) (95% CI = 9.05–29.97%; 95% CI = 19.42–43.98%) (Table 1, Figure 1). All three districts of Submountain Zone are at higher risk indicating the impact of biotic and abiotic factors on the incidence of disease in relation to the disease incidence. Temperature and humidity are well known crucial factors for the development of major vector* B. microplus*, and the climate factor seems to be determinant for the distinct epidemiological conditions found in the semihumid region of Submountain Zone of Punjab. The total normal rainfall of the Kandi region varies from about 800 to 1500 mm, about three-fourths of which is received during rainy season in a few rainy days (http://dolr.nic.in/dolr/downloads/spsp/SPSP-Punjab.pdf). The parasitological and molecular prevalence of* A. marginale* revealed in this study is closed to a previous study conducted in Punjab [4]. The areas covered in the present study have comparatively higher prevalence of* B. microplus* [8] as an effect of favorable macroclimatic factors, thus posing higher threats of anaplasmosis in the area.

**Figure 2:**
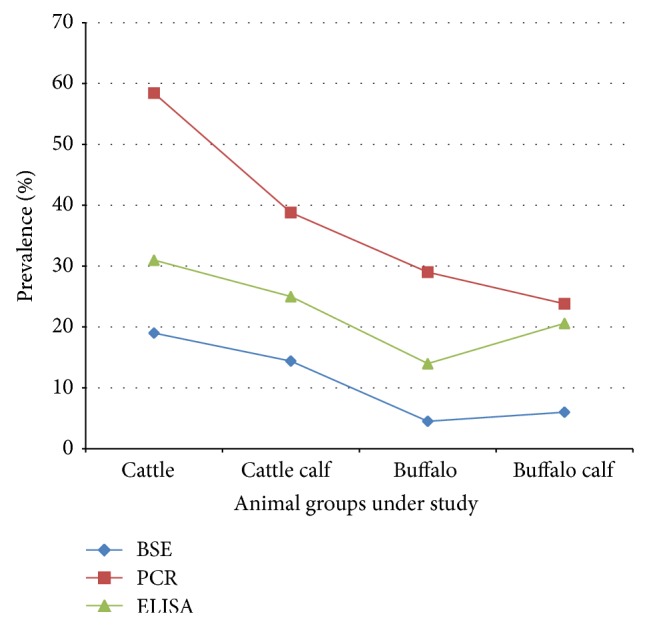
Incidence of* A. marginale* among different animal groups under study as diagnosed by Blood Smear Examination (BSE), Enzyme Linked Immunosorbent Assay (ELISA), and Polymerase Chain Reaction (PCR) revealing cattle as the most exposed group.

Between the two species under study, cattle were shown to have higher susceptibility/exposure to the disease as revealed by serological (28.68%; 95% CI = 21.95–35.41%) as well as molecular test (49.61%; 95% CI = 11.23–35.82%). The incidence of disease varied significantly among the four animal groups with the PCR, highest being in cattle adults (58.11%) (95% CI = 48.41–67.80%) and lowest being in buffalo calves (23.57%) (95% CI = 11.23–35.82%) (Table 1, Figure 2). However, by microscopy and ELISA, there existed nonsignificant difference in the incidence of disease among the four animal groups. Cattle showed highest incidence of disease both by microscopy (18.92%) (95% CI = 11.22–26.61%) and ELISA (31.08%) (95% CI = 21.98–40.17%); however, study explicates the role of buffalo as the potent carrier animal by not exhibiting any clinical symptoms but still harbouring the infection. Cattle displayed a higher incidence of disease than buffaloes by all the three diagnostic techniques. Cattle were found to be more prone to anaplasmosis infection as compared to their calves. Similar trend was seen among buffaloes as revealed by PCR; however, the detection based on microscopy and ELISA depicted higher incidence of the disease in buffalo calves (Figure 2). Significant difference in the seroprevalence of* A. marginale* among different groups [9] may be influenced by factors such as race, age, and physiological and immunological status. It is noteworthy to mention that parasite inoculation rate by other biological vectors [10–12] and other sources of mechanical transmission, namely, blood-sucking dipterans and fomites [13] may also affect the enzootic stability of any geographic area when* A. marginale* is prevalent. Additionally, the lowest seropositivity (*p* < 0.05) seen in cattle aged up to 12 months can be explained by immune protection due to colostral antibodies and/or low rate of inoculation of* A. marginale* by the vectors as the animals in this age group have much lower contact with the vectors compared to older ones [14].

**Figure 3:**
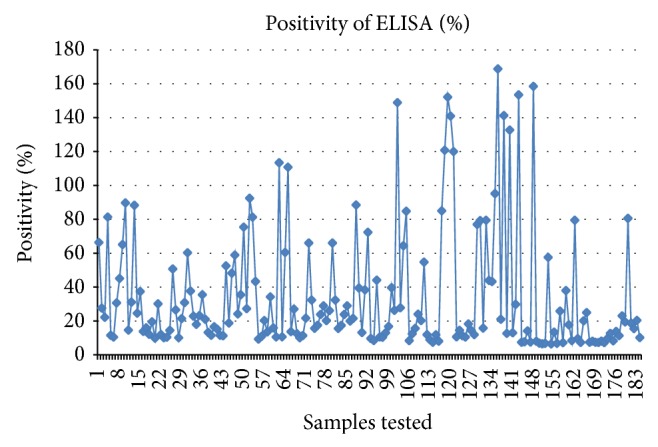
Percent positive plot of samples tested for anti-*A. marginale* antibody by indirect ELISA.

**Figure 4:**
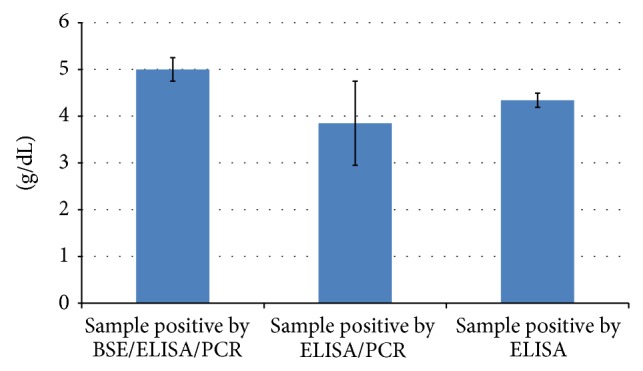
Serum globulin level in three groups tested positive by Blood Smear Examination (BSE), Enzyme Linked Immunosorbent Assay (ELISA), and Polymerase Chain Reaction (PCR).

The positivity by the three techniques markedly differed revealing percent positivity of 13.58% (*n* = 25) (95% CI = 9.31–17.85%) by microscopy, 25.54% (*n* = 47) (95% CI = 20.11–30.97%) by ELISA, and 42.39% (*n* = 78) (95% CI = 36.23–48.54%) by PCR (Table 2). Out of the 184 sample tested, 12 were positive and 77 were negative by all the three tests indicating 48.36% concordance between these tests (Table 2). Forty-nine samples were exceedingly positive by PCR indicating its higher sensitivity over microscopy and ELISA. Twenty samples negative by PCR could be detected positive by ELISA which indicated persistence of antibodies after clearance of infection, while 57 animals which were detected positive by PCR and negative by ELISA may indicate recent infection or may be carrying too low parasitemia to produce detectable antibodies. Out of the 47 samples positive by ELISA, the percent positive of serum antibody in animals positive by all the three tests (*n* = 12) was seen to be more than 100% (Figure 3), which was also corroborated with high globulin levels (Figure 4).

The assessment of risk factor with results of PCR technique as the reference indicates that cattle (Odd's ratio = 2.884, 95% CI = 1.364–6.165%), particularly those of age > 1 years (Odd's ratio = 2.204, 95% CI = 1.161–4.198%) of district Pathankot (Odd's ratio = 3.182, 95% CI = 1.341–7.656%) of Submountain Zone (Odd's ratio = 2.086, 95% CI = 1.81–4.042%) are at high risk of anaplasmosis caused by* A. marginale*.

**Figure 5:**
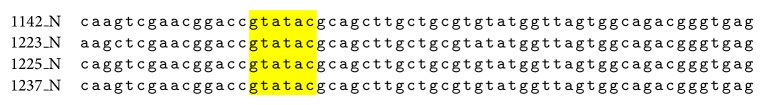
Multiple Sequence Alignment of the four samples positive by 16S rRNA nPCR showing* A. marginale* specific restriction site (highlighted are the restriction sites GTATAC).

Of twenty-five samples positive in slide examination, five cases were there which could not be detected positive by msp1*β* PCR, but 4 of those could show positive amplification by 16S rRNA nPCR specific for* Anaplasma* spp. However, RFLP specific for* A. marginale* could not give conclusive results; that may be due to less concentration of PCR product or small difference in the size of PCR product and restriction digestion product. All four sequenced products revealed the presence of* A. marginale* specific restriction site (GTA↓TAC) in all the samples (Figure 5).

**Figure 6:**
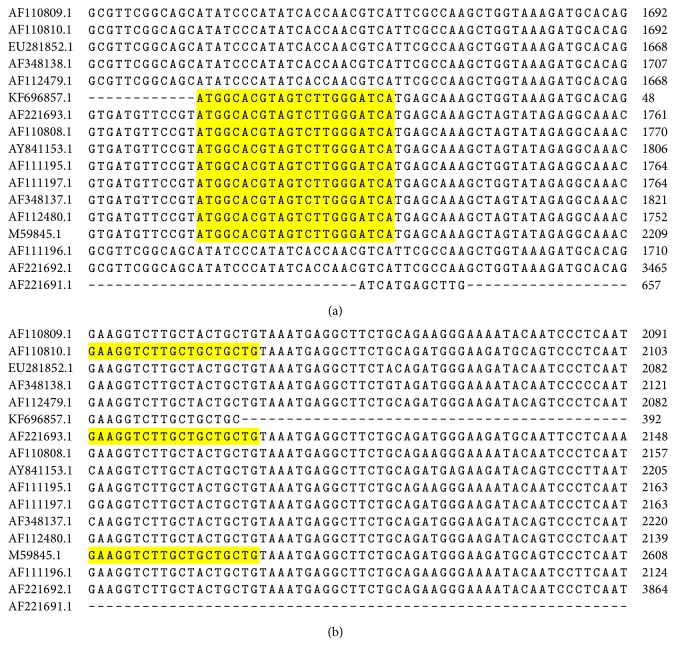
(a) Multiple sequence alignment of various geographic isolates of* A. marginale* showing variations at forward primer binding site of msp1*β* used in the present study. (b) Multiple sequence alignment of various geographic isolates of* A. marginale* showing variations at reverse primer binding site of msp1*β* used in the present study.

**Figure 7:**
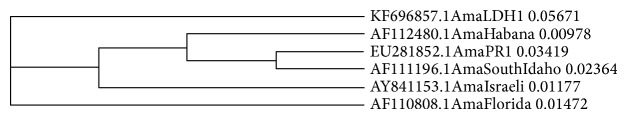
Phylogenetic relationship of local strain (KF696857.1) for the partial msp1b coding sequences of different strains of* Anaplasma marginale*.

There may be a possibility that the msp1*β* PCR showed negative results due to the polymorphism among geographic isolates of* A. marginale* [15] which was also seen in* A. marginale* Str. Florida (AF110809.1), Str. PR1 (EU281852.1), and Str. South Idaho (AF111196.1) (Figures 6(a) and 6(b)), the strain closely related to our local strain submitted under accession number KF696857 (previous study) (Figure 7).

Due to the limitation of degradation of mounted blood smear, one sample still negative by PCR could not be confirmed for* A. marginale* upon rechecking.

With PCR as the reference standard (*n* = 82), the sensitivity and specificity of microscopy (*n* = 25) were found to be 29% and 99% percent, while indirect ELISA targeting gene encoding 19 kD protein (*n* = 47) showed sensitivity and specificity to be 32% and 79%, respectively. There was a fair agreement between microscopy and PCR (Kappa = 0.304, 95% CI = 0.202–0.326%) while a slight agreement between ELISA and PCR (Kappa = 0.093, 95% CI = −0.049–0.231%), which may be because of antigen cross reactivity; thus, serological tests do not produce reliable results [16–18].

The results of the current study demonstrate the possible variation in the msp1*β* gene among the isolates of Punjab and also depict great transmission potential of* A. marginale* in the area targeted. MSPs as important tools for recombinant protein, monospecific and monoclonal antibodies, isolate variability, and potential value in diagnostic assays and vaccines [15], thus the present study may provide future directions for control measures. These data may provide valuable input to managers of livestock and can help understanding the status of herds as well as for planning future interventions strategies. Further the findings of molecular prevalence of* A. marginale* indicated that cattle, particularly those of age >1 year of district Pathankot of Submountain Zone, are at high risk of anaplasmosis caused by* A. marginale*, while the overall incidence of the disease was associated with the geographic location of the areas under study.

## Figures and Tables

**Table 1:** Incidence of *A. marginale* among different districts and animal groups under study as diagnosed by Blood Smear Examination (BSE), Enzyme Linked Immunosorbent Assay (ELISA), and Polymerase Chain Reaction (PCR).

Factors	Total	BSE		ELISA		PCR		Odd's ratio	
		Positive (%)	95% CI	Positive (%)	95% CI	Positive (%)	95% CI	(In terms of PCR)	95% CI
Zones									
Submountain Zone	109	16 (14.67)	8.95–20.41	27 (24.77)	17.78–31.75	54 (49.54)	41.44–57.63	2.086	1.81–4.042
Undulation Zone	75	9 (12.0)	5.65–18.34	20 (26.67)	18.03–35.29	24 (32)	22.89–41.10	0.479	0.247–925
Province									
Gurdaspur	41	8 (19.51)	9.05–29.97	13 (31.71)	19.42–43.98	16 (39.02)	26.14–51.89	0.836	0.387–1.797
Hoshiarpur	36	6 (16.66)	6.17–27.16	6 (16.67)	6.17–27.16	17 (47.22)	33.16–61.28	1.276	0.712–2.819
Pathankot	32	2 (6.25)	−0.98–13.48	8 (25.0)	12.06–37.93	21 (65.62)	51.43–79.81	3.182	1.341–7.656
Rupnagar	35	3 (8.57)	0.57–16.56	9 (25.71)	13.23–38.19	13 (37.14)	23.34–50.94	0.764	0.334–1.732
Nawanshahr	40	6 (15.0)	5.45–24.54	11 (27.5)	15.56–39.43	11 (27.50)	15.56–39.43	0.436	0.188–0.995
Species									
Cattle	129	22 (17.05)	11.46–22.65	37 (28.68)	21.95–35.41	64 (49.61)	42.17–57.05	2.884	1.364–6.165
Buffalo	55	3 (5.45)	0.27–10.63	10 (18.18)	9.39–26.97	14 (25.45)	15.53–35.38	0.347	0.162–0.733
Age									
>1 year	95	15 (15.78)	9.46–22.11	26 (27.36)	19.64–35.09	49 (51.57)	42.92–60.24	2.204	1.161–4.198
<1 year	89	10 (11.23)	5.57–16.89	21 (23.59)	15.98–31.20	29 (32.58)	24.18–40.98	0.454	0.288–0.862

**Table 2:** Correlating between the findings of Blood Smear Examination (BSE), Enzyme Linked Immunosorbent Assay (ELISA), and Polymerase Chain Reaction (PCR).

		Polymerase chain reaction (PCR)			
		Positive	Negative		
Blood Smear Examination (BSE)	Positive	12^abc^	2^bc^	Positive	Enzyme linked immunosorbent assay (ELISA)
	Negative	13^ac^	20^c^	Positive	
	Positive	8^ab^	3^b^	Negative	
	Negative	49^a^	77^d^	Negative	

Superscript “a” indicates samples positive by PCR, “b” indicates samples positive by blood smear examination, “c” indicates samples positive by ELISA, and “d” indicates samples negative by all three testes.
